# Lipid Peroxidation in Cancer Therapy: Molecular Mechanisms Involving Oxidative Stress, Cell Death, and Therapeutic Response

**DOI:** 10.3390/molecules31122072

**Published:** 2026-06-12

**Authors:** Wiktoria Andryszkiewicz, Zuzanna Cichowska, Michał Filipski, Kamila Szyda, Anna Wietrzyk, Piotr Szpak, Julita Kulbacka

**Affiliations:** 1 Faculty of Medicine, Wroclaw Medical University, Pasteura 1, 50-367 Wroclaw, Poland; wiktoria.andryszkiewicz@student.umw.edu.pl (W.A.); zuzanna.cichowska@student.umw.edu.pl (Z.C.); kamila.szyda@student.umw.edu.pl (K.S.); anna.wietrzyk@student.umw.edu.pl (A.W.); 2 University Hospital in Wroclaw (USK), Borowska 213, 50-556 Wroclaw, Poland; michal.fili@outlook.com; 3 Department of Molecular and Cellular Biology, Faculty of Pharmacy, Wroclaw Medical University, Borowska 211A, 50-556 Wroclaw, Poland; piotr.szpak@umw.edu.pl; 4 Department of Immunology and Bioelectrochemistry, State Research Institute Centre for Innovative Medicine, LT-08406 Vilnius, Lithuania

**Keywords:** lipid peroxidation, reactive oxygen species, ferroptosis, GPX4, SLC7A11, combination therapy, immunogenic cell death, cuproptosis

## Abstract

Lipid peroxidation (LPO) is a process where polyunsaturated fatty acids (PUFA) in cellular membranes are oxidized. This process is mediated by reactive oxygen species (ROS) and leads to the formation of reactive products, including 4-hydroxynonenal (4-HNE), malondialdehyde (MDA), and oxidized phospholipids. At low concentrations these products act as second messengers in adaptive redox signalling and metabolic homeostasis, whereas at higher concentrations they compromise membrane integrity and promote cell death. Lipid peroxidation plays a crucial role in anticancer therapies. Here we focus on three mechanistically complementary drugs—sorafenib, cisplatin, and olaparib—because each converges, directly or indirectly, on the redox/LPO axis (system xc−/GPX4 modulation, mitochondrial ROS, and SLC7A11 regulation, respectively), modulating tumor cell responses by inducing PUFA oxidation, mitochondrial dysfunction, and membrane damage. However, tumor cells have several protective pathways against oxidative stress, such as increased expression of glutathione peroxidase 4 (GPX4), the SLC7A11 system Xc, and detoxification of reactive aldehydes. Enrichment of membranes with PUFA increases susceptibility to lipid peroxidation and ferroptosis, thereby sensitizing tumor cells to therapy, whereas enrichment with monounsaturated fatty acids (MUFA), driven by the SREBP1–SCD1 axis, limits peroxidation and confers resistance. Among regulated cell death modalities, ferroptosis is strictly dependent on lipid peroxidation, whereas apoptosis, necrosis, necroptosis, pyroptosis, and immunogenic cell death can be modulated by lipid peroxidation but do not universally require it. Collectively, these mechanisms indicate that lipid peroxidation is an important—though not exclusive—determinant of anticancer drug sensitivity and resistance, and that its dual, context-dependent role (tumor-suppressive at high flux, tumor-promoting under chronic, sub-lethal exposure) must be considered when designing LPO-based therapeutic strategies.

## 1. Introduction

Lipid peroxidation plays a significant role in various physiological and pathological mechanisms. It is a multistep process in which oxygen-free radicals or nonradicals generate lipid peroxides through oxidative degradation by targeting polyunsaturated fatty acids (PUFAs) with carbon-carbon double bonds. Moreover, glycolipids, phospholipids (PLs), and cholesterol can be susceptible to potentially lethal peroxidative modifications [1,2,3].

Our review is organized around a single conceptual axis: how lipid peroxidation acts as a node that links oxidative stress to regulated cell death and, ultimately, to therapeutic response and resistance in cancer. We first summarize the chemistry and major products of lipid peroxidation only to the extent needed to interpret the therapeutic literature, then examine three clinically used drugs that engage this axis through distinct upstream mechanisms (sorafenib, cisplatin, olaparib), the principal resistance pathways that tumors use to restrain peroxidation, and the cell-death programs that lipid peroxidation regulates to differing degrees.

Lipid peroxidation, ferroptosis, and oxidative stress in cancer have each been reviewed extensively. The specific contribution of this review is to integrate three usually separate strands—(i) the drug-level pharmacology of LPO-engaging agents, (ii) the antioxidant resistance network (NRF2, GPX4, FSP1–CoQ10, SLC7A11, ALDH, and the under-discussed enzyme paraoxonase-2, PON2), and (iii) the differential dependence of regulated cell-death modalities on lipid peroxidation into one explanatory framework, while explicitly distinguishing established mechanisms from associative or context-dependent ones. We also focused on the dual, pro- and anti-tumor nature of lipid peroxidation, which is frequently under-emphasized in mechanism-focused reviews.

### 1.1. Search Strategy and Study Selection

Relevant literature was identified by searching PubMed/MEDLINE, Scopus, and Web of Science for articles published up to 2026, using combinations of the terms “lipid peroxidation”, “ferroptosis”, “4-HNE”, “malondialdehyde”, “GPX4”, “SLC7A11/xCT”, “FSP1/AIFM2”, “ACSL4”, “SCD1/SREBP1”, “NRF2”, “cancer therapy”, “chemoresistance”, and the individual drug names (sorafenib, cisplatin, olaparib). Priority was given to original mechanistic studies, recent (2020–2025) primary reports, and authoritative consensus/nomenclature documents. Reviews were used mainly to trace primary sources. Non-English records, conference abstracts without peer review, and reports lacking mechanistic data on lipid peroxidation were excluded. Because this is a narrative rather than a systematic review, no formal meta-analytic synthesis or risk-of-bias scoring was performed; this is acknowledged as a limitation, and selection was guided by mechanistic relevance to the LPO–cell death–therapy axis.

The structural complexity of membrane phospholipids is largely determined by the composition and positional distribution of their acyl chains: the sn-1 position is typically enriched in saturated fatty acids (SFAs) and monounsaturated fatty acids (MUFAs), whereas the sn-2 position is often enriched in polyunsaturated fatty acids (PUFAs). In SFAs, an increased rigidity is noticed, which provides better protection against harmful lipid peroxidation and cell death [2,4]. In contrast, PUFAs are highly susceptible to lipid peroxidation mediated by oxygen-derived free radicals, leading to the formation of lipid peroxyl radicals (PLOO•) and lipid hydroperoxides (PLOOH). Lipid peroxyl radicals can further propagate the chain reaction by removing hydrogen atoms from neighboring lipids, generating reactive products that participate in subsequent reactions. This process ultimately compromises cell membrane integrity and may lead to cell death [1,2,5]. Lipid peroxidation generally proceeds through three main stages: initiation, propagation, and termination [6].

### 1.2. Initiation

**Figure 1:**
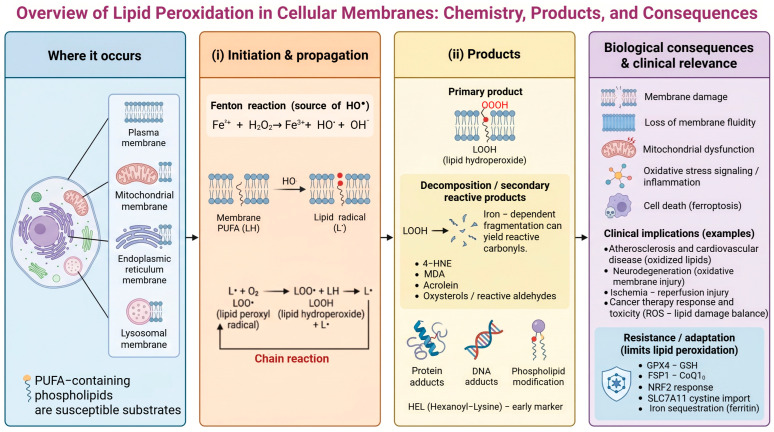
Overview of lipid damage in cell membranes. The diagram shows lipid damage in PUFA-rich phospholipids of plasma, mitochondrial, endoplasmic reticulum, and lysosomal membranes. It explains: (i) how radicals start and spread the damage; (ii) formation of lipid hydroperoxides (LOOH) as first products; (iii) breakdown of LOOH into other reactive substances, including 4-HNE, MDA, acrolein, oxysterols, and other reactive aldehydes; and (iv) later biological effects, such as membrane damage, reduced membrane flexibility, mitochondrial problems, oxidative stress signals, inflammation, protein and DNA damage, ferroptotic cell death, and tissue injury important for health. Defense systems reduce lipid damage through GPX4, GSH, FSP1, CoQ10, NRF2 response, SLC7A11-controlled cystine uptake, and ferritin-based iron storage. Created in BioRender. Kulbacka, J. (2026) https://BioRender.com/s5yttrs.

Initiation is an important process that generates a lipid radical (L•), which is crucial in subsequent steps of lipid peroxidation (Figure 1). This process is triggered via nonenzymatic pathways (e.g., external physical and chemical factors such as air pollution, smoking, ultraviolet (UV) light, or ionizing radiation) and enzymatic pathways (e.g., NADPH oxidase, xanthine oxidase, uncoupled nitric oxide synthase, and cytochrome P450). Additionally, free radicals are generated during the mitochondrial electron transport chain. In vitro, PUFA oxidation can be induced by metal ions (e.g., iron, copper), enzymes, hydroxyl radical, or gamma irradiation [6]. Moreover, the nonenzymatic pathway referred to as “nonenzymatic phospholipid autoxidation” may be controlled by redox-active metals, with labile, highly reactive iron playing a major role. In this process, called “Fenton reaction” a hydroxyl radical (HO•) is produced to initiate the lipid peroxidation [2].

(Fe^2+^ + H_2_O_2_→ Fe^3+^ + HO^•^ + OH^−^)

### 1.3. Propagation

In the propagation phase, molecular oxygen rapidly reacts with the carbon-centered radical L• to form a peroxyl radical (LOO•). During radical chain oxidation, a hydrogen atom from a neighboring lipid substrate molecule is removed, leading to the formation of a new lipid radical L• and lipid hydroperoxide (LOOH). This continues the chain reaction. In an analogous way, the phospholipid hydroperoxides (PLOOH) can also be transformed into o lipid hydroxy radical (PLO•) and PLOO•. The propagation of chain reactions continues until the production of substrates that are necessary for the termination process [2,3,6].

### 1.4. Termination

Termination is the final stage of lipid peroxidation. It occurs through two principal mechanisms: (i) radical–radical recombination, in which two chain-carrying radicals combine to form non-radical products, and (ii) interception of chain-carrying peroxyl radicals by radical-trapping antioxidants (RTAs) such as α-tocopherol (vitamin E), which donate a hydrogen atom to LOO• and are themselves converted into comparatively unreactive, resonance-stabilized radicals. In both cases, the chain is broken rather than “inhibited by radicals”. During this process, non-radical products are created [5,7,8].

## 2. Lipid Peroxidation Products

The wide variety of products generated during lipid peroxidation chain reactions exhibits high biological activity and can damage DNA, proteins, and enzymes. Furthermore, these products may promote signaling pathways that lead to cell death [5,8]. It is useful to distinguish primary products (lipid hydroperoxides, LOOHs/PLOOHs), which are the direct radical-chain intermediates, from secondary products generated by their decomposition—reactive aldehydes such as MDA, 4-HNE, acrolein, and the cyclized isoprostanes/isoketals (Figure 1)—because the two classes differ in stability, diffusibility, and downstream biology. Among the secondary products from lipid peroxidation can be distinguished, including fragmentation products from non-enzymatic oxidation: e.g., malondialdehyde (MDA), propanal, hexanal, acrolein, and 4-hydroxynonenal (4-HNE); as well as cyclization products from enzymatic reactions: e.g., prostaglandin endoperoxides, isoprostanes (IsoP), isoketales [1,3,8,9]. Recently, Nε-hexanoyl-lysine (HEL) has also been identified as a novel biomarker that is specifically generated from the oxidation of omega-6 polyunsaturated fatty acids, expanding the repertoire of known lipid peroxidation products [10]. MDA and 4-HNE appear to be among the two most frequently tested electrophiles. MDA is recognized as one of the major mutagenic substances produced in the process, while 4-HNE is the most toxic compound [3,6,11].

4-HNE is a widely studied target and has been established as a highly reactive and cytotoxic α,β-unsaturated aldehyde. Produced by the degradation of arachidonic acid (AA) and other long-chain PUFAs through both enzymatic and nonenzymatic mechanisms [3]. The impact on cells depends mainly on the extent of lipid peroxidation and the amount of reactive aldehydes formed. Therefore, 4-HNE may act either in normal cellular physiology or in pathological processes [12]. For the measurement of the amount of 4-HNE, immunoblotting or immunohistochemistry can be used [3,5]. Because of its strong electrophilic properties, it forms adducts with DNA, proteins, and lipids, which could lead to their damage [6,10]. During these processes, 4-HNE plays a significant role in signaling pathways and exhibits cytotoxic effects, primarily due to its ability to covalently modify macromolecules [3].

The signaling properties include the regulation of the heat shock response, endoplasmic reticulum (ER) stress, NF-kB signaling, and the DNA damage response, as well as antioxidant pathways such as the Keap1/Nrf2 signaling axis (Figure 1) [6]. Moreover, at basal levels of antioxidant enzyme activity, 4-HNE detoxification may be insufficient; however, cells may still survive due to 4-HNE-mediated regulation of various transcription factors. Those factors are sensitive to stress, particularly Nrf2, AP-1, NF-κB, and peroxisome-proliferator-activated receptors. Multiple studies have shown that 4-HNE-dependent activation of Nrf2 is a major regulator of oxidative stress. By use of the Nrf2-antioxidant-response element activators, it is possible to protect from 4-HNE cytotoxicity. Therefore, 4-HNE is considered one of the most significant bioactive markers of lipid peroxidation alongside MDA [3,13]. MDA, an electrophilic aldehyde, is also one of the most widely used and reliable biomarkers of lipid peroxidation of omega-3 and omega-6 fatty acids.

Its activity depends on pH, where physiological pH leads to reduced chemical reactions, whereas low pH results in enhancing its reactivity, and MDA acts as β-hydroxyacrolein [3,14]. Furthermore, MDA adducts are involved in numerous secondary deleterious reactions, including crosslinking [3]. MDA exhibits a strong affinity to form adducts with DNA. They are responsible for inducing mutations in biomolecules and biochemical traits. Additionally, MDA may be associated with chronic diseases and with aging. Because of elevated MDA amount in the cytoplasm, it can create adducts with proteins through Nε- (2-propenal) lysine or 1-amino-3-iminopropene-type and pyridyl-dihydropyridine-type lysine-lysine cross-links. The response to exposure to cigarette fumes or alcohol is a hallmark favoring the formation of toxic adducts. The presence of products such as MDA-protein and MDA-acetaldehyde-protein is associated with a pro-inflammatory response in the body [3,9].

Due to its reactivity with thiobarbituric acid (TBA), MDA can be determined using the TBA assay coupled with UV-visible spectrometry. In this reaction, a highly pigmented chromogen and a fluorescent red adduct are formed. Other analytical techniques developed over the past decade for the detection of free and total MDA, such as gas chromatography-mass spectrometry (GC-MS/MS) and liquid chromatography-mass spectrometry (LC-MS/MS), have also proven useful [3,5,14].

IsoP are stereoisomers, prostaglandin-like substances present in biological fluids (blood, urine, cerebrovascular fluid). They can be formed via oxidative cyclization from unstable prostaglandin H2-like cyclic endoperoxide intermediates. IsoP are either converted to F2-isoprostanes (F2-IsoPs) or rearranged form E2/D2-isoprostanes (E2/D2-IsoPs). This stage relies on lipid peroxidation of PUFAs, e.g., arachnoid acid (AA), independently of cyclooxygenase [5,9,15]. The most characteristic role of IsoP is to modulate inflammatory pathways. Notably, F2-IsoPs are strong vasoconstrictors, participate in platelet activity, suppress angiogenesis, and contribute to atherosclerosis due to their adhesion-stimulating properties, especially in monocytes and neutrophils. A highly reactive product from isoprostanoids metabolism, such as isoketals (IsoKs) may modify the proteins` structure and produce toxic adducts with phosphatidylethanolamine [9]. In gas chromatography/mass spectrometry (GC/MS) with negative ion chemical ionization, the lipid peroxidation-derived 5- and 15-F2t isoprostanes can be used to accurately quantify their concentrations in biological fluids [5].

Oxidized phospholipids (OxPL) are created mainly during non-enzymatic pathways of lipid peroxidation [16,17]. PUFAs show higher susceptibility to oxidation caused by oxidative stress, compared to saturated fatty acids, due to the located methylene groups between double bonds [18]. Unfortunately, the mechanisms by which neutralizing OxPL and their functions in vivo remain incompletely understood [17]. Created oxidation-specific epitopes, such as oxidized phosphocholine, which is one of the best-described substances in this group. OxPL are commonly characterized by its pro- inflammatory actions [18,19]. Additionally, among the biological activities of OxPLs, the most notable include activation of cell adhesion molecules and stimulation of numerous chemokines (e.g., MCP-1, MCP-3, MCP-5) [16]. Nowadays, studies suggest that therapies that target inactivating OxPL can be helpful in reducing inflammation and even in the progression of atherosclerosis and aortic stenosis [17]. Lipidomics is a novel field that studies cellular lipid metabolism. Its main activities involve analytical chemistry, mass spectrometry, and the collection of biological samples and experimental data [20]. Lipids often work as mediators, and lipidomic signatures work as highly sensitive markers. They enable the detection of a variety of pathologies and structural changes, as well as the prediction of diagnosis, prognosis, and treatment response [21]. Oxidative lipidomics enables the quantification of specific PLs and their hydroperoxides [20]. This technique enables the detection of products from various processes and generates lipidomic patterns [21].

## 3. Effects of Lipid Peroxidation on Anticancer Drug Efficacy

Cancer is nowadays one of the most common diseases. Most treatment strategies target molecular mechanisms that disrupt DNA damage checkpoints, activate non-receptor tyrosine kinases, or regulate the hedgehog signaling pathway. However, more recently, new ideas have been suggested. Further understanding and exploration of lipid metabolism may be crucial, especially in the case of lipid peroxidation and ferroptosis—an iron-related programmed cell death in anti-tumor treatment [22,23]. Anticancer effects can be promoted by various chemotherapeutic agents through direct or indirect induction of lipid peroxidation [24]. Iron overload, however, can cause an unpredictable increase in toxic oxidative products in cancer-free cells. Thus, any ferroptosis inducers must be administered carefully. Studies have shown an indirect correlation between the use of pharmacological inhibitors and the induction of ferroptosis [22,25]. Sorafenib, cisplatin, and olaparib are discussed together below not because they share a single mechanism, but because they illustrate three different entry points into the LPO axis; their primary, label mechanisms remain kinase inhibition, DNA cross-linking, and PARP inhibition, respectively.

Sorafenib, used in hepatocellular carcinoma (HCC), is primarily an oral multikinase inhibitor (targeting RAF, VEGFR, PDGFR, and others) that suppresses tumour proliferation and angiogenesis; its ability to trigger ferroptosis is a secondary, context-dependent property rather than its principal or universal mechanism of action. At the same time, it promotes apoptosis in cancer cells by inducing oxidative stress and accumulating reactive oxygen species (ROS) products [1,24,26,27,28]. In the contexts where sorafenib does induce ferroptosis, the most consistent mechanism is inhibition of the cystine–glutamate antiporter system xc− (SLC7A11), which depletes intracellular cysteine and glutathione (GSH) and thereby limits GPX4 activity, allowing lipid hydroperoxides to accumulate; a direct, high-affinity inhibition of GPX4 by sorafenib is not well supported, and ferroptotic responses vary substantially between cell lines [1,29,30,31]. Suppression of critical molecules involved in ferroptosis, cystine/glutamate antiporter, and glutathione peroxidase may lead to the eradication of chemotherapy/radiotherapy-resistant tumor cells [32]. Cisplatin is an antineoplastic medication. It serves as the first-line chemotherapy for several solid tumors. Unfortunately, the therapeutic possibilities are frequently limited due to its resistance and the variety of mechanisms involved in it, especially in head and neck cancer [33]. Mechanistically, the effects of cisplatin on the LPO axis should be separated into distinct layers that are often conflated. First of all, cisplatin’s canonical, dominant mechanism is the formation of DNA intra- and interstrand cross-links that trigger the DNA damage response and apoptosis. Next, downstream of mitochondrial injury, cisplatin elevates mitochondrial ROS, which can, in turn, drive lipid peroxidation. Cisplatin can also modulate autophagy, and in some models it promotes GPX4 degradation and lipid peroxidation dependent ferroptosis. These layers are mechanistically separable, and lipid peroxidation is best regarded as one downstream consequence of cisplatin-induced oxidative stress rather than its primary action. In the other study shown, cisplatin treatment amplifies autophagosome production and promotes autophagosome-lysosome fusion by recruiting RAB7 to autolysosomes, thereby mobilizing the cancer stem cell population. Furthermore, this study indicates that blocking cisplatin-stimulated flux in oral CD^44+^ cells decreases cancer probability and induces apoptosis in highly resistant cancer stem cells by triggering ROS production. Normally, NRF2 signaling restricts mitochondrial superoxide generation in autophagy-deficient oral CD^44+^ cells treated with cisplatin. Therefore, combined inhibition of autophagy and NRF2 signaling enhances cisplatin chemosensitivity. Moreover, elevated mitochondrial ROS levels are associated with reduced cisplatin resistance [34].

Olaparib belongs to the poly (ADP-ribose) polymerase (PARP) inhibitors. They are used in the treatment of platinum-sensitive ovarian cancer; the effectiveness in cases with platinum-resistant ovarian cancer is restricted [35]. Its principal mechanism is PARP trapping and the synthetic-lethal accumulation of DNA damage in homologous recombination–deficient cells. The connection to lipid peroxidation is mechanistically coherent: olaparib can down-regulate the cystine transporter SLC7A11 in a p53-dependent manner, lowering GSH and sensitizing cells to ferroptosis, and it can thereby enhance the activity of ferroptosis inducers. A recent study investigated the effects of combination therapy with olaparib and arsenic trioxide (ATO). The combination of high-dose ATO with olaparib can be beneficial in many solid tumors by inducing oxidative stress and causing DNA damage. This process ultimately leads to apoptosis [36]. Despite the therapeutic benefits of olaparib, resistance to this PARP inhibitor may develop through autophagy induction. This process results in increased availability of free fatty acids [37]. This last point provides the logical bridge to PUFA biology: the free fatty acids mobilized during olaparib resistance include peroxidation-prone PUFAs, whose handling determines whether the cell is sensitized to, or protected from, ferroptosis. Eicosapentaenoic acid (EPA) belongs to a group of n-3 long-chain fatty acids. Regular intake of EPA has been shown to benefit the whole body, providing protection against cardiovascular disease and exhibiting anti-inflammatory effects. Both n-3 fatty acids and docosahexaenoic acid (DHA) are favorable in interrupting inflammatory processes. Moreover, those molecules are naturally occurring anti-inflammatory and pro-resolving mediators that antagonize pro-inflammatory signaling in chronic diseases [34,35]. Increased PUFAs oxidation may occur when the exposed bis-allylic site of PUFAs is damaged by free radicals. The resulting end products vary depending on the type of oxidized PUFA. Enzymatic oxidation of linoleic acid results in hydroperoxy octadecadienoates (HPODEs), while AA oxidation will create prostaglandins, thromboxanes, leukotrienes, lipoxins, hydroperoxyeicosatetraenoic acid, epoxyeicosatrienoic acid, and 20-hydroxyeicosatetraenoic acid [24].

## 4. Lipid Peroxidation and Chemoresistance

### 4.1. An Integrated Model of Lipid-Peroxidation Resistance

Instead of acting alone, the resistance factors can also work together as an antioxidant network that determines how much lipid peroxidation a cell can handle before it becomes lethal. Cysteine enters the cell through SLC7A11 (system xc−) and helps make glutathione, which is needed by GPX4, the main enzyme that reduces phospholipid hydroperoxides. The FSP1–CoQ10 and GCH1–BH4 systems trap radicals without relying on GPX4. NRF2 helps regulate many of these defenses by increasing the expression of proteins such as SLC7A11, GPX4, and FSP1. ALDH enzymes remove harmful aldehyde byproducts, and PON2 breaks down lipid peroxides at the cell membrane. When tumors are exposed to treatment, they often adapt by increasing their antioxidant defenses and relying more on GPX4 and FSP1, sometimes becoming more mesenchymal and resistant [38]. This is why blocking just one part of the network is often not enough, and why researchers are testing combinations, such as blocking both GPX4 and FSP1. Still, the role of each network component depends on the tumor type and the situation.

### 4.2. Tumor Adaptation

The cellular transduction of the signal that controls processes such as cell proliferation, differentiation, and death can be modulated by lipid peroxidation. Additionally, this process can activate immune cells, thereby impacting their anticancer properties and tumor immunity. This plays a crucial role in shaping the tumor microenvironment (TME) [1], thereby creating new opportunities for the development of anticancer drugs. However, multiple mechanisms of chemoresistance and tumor adaptation continue to limit therapeutic efficacy. Ferroptosis, which is a nonapoptotic cell death mechanism based on the accumulation of lipid ROS and iron dependency, can be associated with drug resistance [36].

### 4.3. NRF2

The transcription factor Nrf2 plays a central role in multiple mechanisms underlying cancer cell survival and adaptation. Overactivation of NRF2 promotes various processes, including initiation, progression, metastasis, and drug resistance. Its role is dual and must be distinguished. In normal cells, transient KEAP1-regulated NRF2 activation is a physiological, cytoprotective response that limits oxidative and electrophilic damage. In many tumors, by contrast, constitutive NRF2 activation—through KEAP1 loss-of-function or NRF2 gain-of-function mutations, or oncogene-driven signaling—locks cells into a high-antioxidant state that promotes initiation, progression, metastasis, and therapy resistance, including resistance to LPO-based therapies. It is this oncogenic, constitutive activation, rather than physiological cytoprotection, that is therapeutically relevant here. Furthermore, the KEAP1-NRF2 axis plays a key role in regulating cellular responses to both electrophilic and oxidative stressors. This pathway in clear cell renal cell carcinoma (ccRCC) is controlled by dipeptidyl peptidase 9 (DPP9). It was discovered that a high level of DPP9 in patients with ccRCC was associated with substantial severity of the tumor and poor prognosis. Hyperexpression of DPP9 leads to NRF2 hyperactivation, thereby suppressing ferroptosis. These processes may contribute to the development of sorafenib resistance in ccRCC cells, suggesting that targeting the lipid peroxidation pathway could represent a potential therapeutic strategy to overcome chemoresistance [35,38]. Pharmacological NRF2 inhibitors (e.g., ML385) and modulators of NRF2 target genes (including FSP1) can re-sensitize tumour cells to lipid peroxidation and ferroptosis.

### 4.4. GPX4 and Drug-Tolerant Persister Cells

Persister cells play a major role in non-mutational drug resistance, as they can survive chemotherapy or targeted therapy. This issue affects a wide range of tumor types, and its underlying mechanisms remain poorly understood. Targeting persister cells may provide an opportunity to prevent relapse in patients [37]. The lipid hydroperoxide-detoxifying enzyme GPX4 converts LOOHs into non-toxic lipid alcohols, thereby preventing ferroptosis. In addition, GPX4 is an essential enzyme in highly mesenchymal, therapy-resistant cancer cells [37,39]. These cells become dependent on GPX4, and loss of its function induces ferroptotic cell death, thereby potentially reducing the risk of tumor relapse. Consistent with these findings, drug-tolerant persister cells that survive front-line therapy often exist in a transient, GPX4-dependent state. GPX4 inhibitors, as well as combined inhibition of GPX4 and FSP1 (as discussed below), can selectively deplete this residual cell population. Co-treatment with GPX4 inhibitors can efficiently deplete the residual persister cell pool; however, sensitivity to these agents varies across cell lines, suggesting that additional factors should be considered [37,39]. 

### 4.5. FSP1 and CoQ10

An additional ferroptosis-suppressing pathway operates independently of the glutathione-dependent GPX4 system, namely the non-mitochondrial CoQ antioxidant pathway. Ferroptosis suppressor protein 1 (FSP1), which was previously known as apoptosis-inducing factor mitochondrial 2 (AIFM2), is recruited to the plasma membrane. Then it reduces coenzyme Q10, generating a lipophilic radical-trapping antioxidant (RTA) that prevents lipid peroxides from propagating. In hundreds of cell lines, increased FSP1 levels correlate positively with resistance to ferroptosis. What is more FSP1 expression is associated with resistance to RSL3, ML210, and ML162, which are GPX4 inhibitors [39]. Since GPX4 and FSP1 work together, blocking just one is often not enough. Recent studies show that high levels of FSP1 make cells resistant to GPX4 inhibitors [38]. Targeting both GPX4 and FSP1 together, for example, by using RSL3 or ML210 with FSP1 inhibitors like iFSP1, may be needed to overcome ferroptosis resistance in persister and mesenchymal cells.

### 4.6. SLC7A11

Cancer stem-like cells also play a significant role in therapy resistance due to their ability to both self-renew and initiate tumor formation. Targeting them could enhance the effectiveness of cancer treatment [40,41]. Stem cell transcriptional factor (SOX2) promotes the transcription of cystine transporter (SLC7A11), which is an essential element of the cystine/glutamate antiporter system xc−. It also regulates cystine uptake, synthesis of glutathione GSH, and resistance to ferroptosis. The activity of SLC7A11 is also controlled by many other factors, such as NRF2 and p53, which regulate its transcription. Agents such as Erastin or Imidazole ketone erastin (IKE) inhibit SLC7A11, thereby reducing intracellular GSH levels and inducing ferroptosis. Other drugs, such as ROS inducers, could decrease SOX2 activity and reduce SLC7A11 activity by oxidizing SOX2, thereby making SOX2-high cancer cells more susceptible to ferroptosis [42]. 

### 4.7. ALDH

Another worth mentioning aspect of tumor resistance to therapy in persister cells is the role of Aldehyde Dehydrogenases (ALDHs). They constitute a group of oxidoreductase enzymes essential for aldehyde detoxification and the oxidation of aldehydes to carboxylic acids. Consequently, ALDHs play a crucial role in stem cell development, differentiation, and maintenance. Their other role is protection against ROS and retinoid acid synthesis. High level of ALDH activity is correlated with poor prognosis for patients and can be present in: lung, prostate, breast, colon, liver, pancreas, stomach, ovary, esophagus, and brain cancer. It has been discovered that ALDH^high^ cells in mantle cell lymphoma are the cause of relapse, and in breast cancer, CD^44+^ ALDH^high^ cells play a role in spreading metastases. Cells with overexpression of ALDH present chemoresistance to many cytotoxic drugs, such as cisplatin, dacarbazine, and cytarabine. A specific ALDH^high^ persister cell subpopulation has stem cell-like characteristics and can switch rapidly between high and low ALDH activity due to its elevated plasticity. ALDH enzymes help remove reactive aldehydes, which reduces the harmful effects of lipid peroxidation. This process connects aldehyde detoxification to the persister phenotype. As a result, researchers are testing ALDH inhibitors with ferroptosis inducers to target persister cells. However, more studies need to be done to understand how they adapt to a high cellular stress environment or survive drug concentrations that are lethal to other cells [37].

### 4.8. Paraoxonase-2 (PON2)

Paraoxonase-2 (PON2) is an antioxidant defense that is often left out of discussions about lipid-peroxidation–based therapy. PON2 is an intracellular, membrane-associated lactonase with antioxidant activity. It is overexpressed in several cancers, such as clear-cell renal-cell carcinoma, triple-negative breast cancer, oral squamous-cell carcinoma, bladder cancer, and melanoma, where it helps maintain redox balance and supports cancer cell survival [43,44]. PON2 can reduce the buildup of lipid peroxides and protect cell membranes from oxidative damage, which may limit cell death caused by lipid peroxidation, including ferroptosis. Because of this, high levels of PON2 may reduce the effectiveness of therapies that depend on lipid peroxidation, make tumors more resistant to ROS-inducing anticancer drugs, and decrease tumor sensitivity to treatments that damage membranes through oxidation. PON2 is part of the tumor antioxidant network, along with NRF2, GPX4, FSP1, SLC7A11, and ALDH, and could be a target for making tumors more responsive to LPO-based therapies.

### 4.9. Tumour-Type Specificity

The importance of these factors is different in each type of cancer. GPX4 is especially important in mesenchymal, therapy-resistant, and persister cancer cells. ACSL4 and PUFA metabolism are key in some epithelial tumors, like colorectal, breast, and prostate cancers, but not in others. SCD1 and MUFA-based protection are common in breast and nutrient-starved pancreatic cancers. The need for FSP1 varies widely across cancer types, and changes in NRF2 or KEAP1 are more common in certain lung and kidney cancers [45]. Because of these differences, it is important to consider the specific cancer type and cell state when talking about “ferroptosis sensitivity.” Using biomarkers to select appropriate treatments will likely be necessary for LPO-based therapies to be used effectively.

## 5. Lipid Remodeling Resistance

Tumor cells can actively or passively remodel lipid metabolism, thereby becoming resistant to various drugs [46]. This remodeling integrates several interacting processes—exogenous fatty-acid uptake (e.g., via CD36), de novo lipogenesis (ACC/FASN), fatty-acid desaturation (SCD1), acyl-chain incorporation and exchange (ACSL and LPCAT/MBOAT enzymes), and lipid-droplet storage—which together set the PUFA-to-MUFA ratio of membranes and hence their susceptibility to peroxidation. Among ferroptosis-related long-chain fatty acids (LCFAs), PUFAs and MUFAs play particularly important roles. PUFAs contain two or more double bonds and are highly susceptible to lipid peroxidation, thereby promoting ferroptosis. In contrast, MUFAs contain only one double bond and are less prone to oxidation by oxygen-derived radicals. Their enrichment in cellular membranes may therefore reduce lipid peroxidation and inhibit ferroptosis [47].

### 5.1. PUFAs

Targeting lipid metabolism might reverse chemo- or radiotherapy resistance across various cancer types [46]. PLs containing PUFAs play an important role in ferroptosis as substrates, generating lipid ROS. In one study, the role of phospholipids (PLs) in enhancing the sensitivity of multiple myeloma (MM) cells to ferroptosis was investigated. MM cells in comparison to diffuse large B cell lymphoma are said to be relatively insensitive to this form of non-apoptotic cell death induced by direct or indirect inhibition of GPX4. It has been shown that PL composition can sensitize MM cells to ferroptosis (via GPX4 inhibition). Those liposomes were packed with RSL3 and prepared from PL-PUFAs, which are often deficient in MM. This study provided promising evidence that lipid nanoparticles may be an efficient way of delivering ferroptosis substrates and inducers to tumor cells. In prostate cancer, GPX4 dependence and ferroptosis hypersensitivity in persister cells have been associated with extensive lipid remodeling, including increased lipid uptake and PUFA enrichment of membrane lipids. Lipase and fatty acid desaturase activities are crucial for persister cells to develop a reliance on GPX4 [46]. Moreover, in colorectal cancer, LPCAT2-mediated lipid droplet (LD) formation might be the solution to restore cellular chemosensitivity. Higher levels of free PUFAs and enhanced esterification of membrane phospholipids lead to lipid peroxidation and play a role in ferroptosis of cancer cells. It also modulates tumor chemosensitivity, which creates additional chemotherapeutic potential. In colorectal cancer, PUFAs contribute to enhanced susceptibility to 5-fluorouracil. In breast cancer, they are used in combination with celecoxib and tamoxifen, and in lung cancer with cisplatin [46].

### 5.2. MUFA via SCD1

Another mechanism of chemoresistance observed in breast cancer involves MUFA production driven by the SREBP1-SCD axis. SFA are converted into oleic acid, which protects tumor cells from ferroptosis. Inhibition of SCD1 is associated with increased sensitivity to ferroptosis; conversely, its upregulation correlates with a poor prognosis in patients. MUFAs are activated by ACSL3 via membrane-bound O-acyltransferases MBOAT1/2 for incorporation. MUFA-CoAs are transferred by MBOAT1/2 into PLs, which reduces peroxidizable substrates. High expression of MBOAT1 occurs in breast cancer and is regulated by ER signaling [48]. Importantly, this axis is also nutrient-regulated: in pancreatic cancer, nutrient deprivation activates the mTOR–SREBP1–SCD1 axis, increasing MUFA synthesis and conferring ferroptosis resistance, and rapamycin suppresses SCD1 and restores lipid peroxidation. Accordingly, combination therapy with sorafenib with rapamycin has been reported to reverse this MUFA-dependent resistance, illustrating how metabolic and redox-targeted agents can be rationally combined [49].

### 5.3. ACSL and LPCAT

Acyl-CoA synthetases long-chain (ACSLs) catalyze the conversion of long-chain fatty acids (LCFAs) into LCFA-acyl-coenzyme A CoA, which is useful in processes such as oxidation of fatty acids, synthesis of lipids, and acylation of proteins. Among those enzymes, ACSL4 plays a decisive role in ferroptosis. ACSL 4 incorporates PUFAs into PLs via lysophosphatidylcholine acyltransferase LPCAT3 [50]. In this way, oxidizable polyunsaturated fatty acid-containing phospholipids (PUFA-PLs) are generated. This leads to peroxidation of PUFA-PLs by lipoxygenase LOX, cytochrome P450 oxidoreductase POR, or phosphatidylethanolamine binding protein 1 (PEBP1). ACSL4 can be the cause of resistance to various drugs such as cisplatin, emodin, doxorubicin, sorafenib, and paclitaxel. Moreover, insertional mutations in ACSL4 have been observed to confer resistance to cell death by inhibiting GPX4 in the myeloid leukemia cell line KBM7. In contrast, both ACSL1 and ACSL3 have been reported to enhance the efficacy of imatinib in chronic myeloid leukemia and simvastatin in non-small cell lung cancer [44]. LPCAT-mediated chemoresistance has also been described. For example, in colorectal cancer, LPCAT2 promotes LD formation, thereby contributing to drug resistance [48]. In addition to ACSL4, the related enzymes ACSL3 and the membrane-bound O-acyltransferases MBOAT1 and MBOAT2 work on the protective side by directing MUFAs into phospholipids. In contrast, ACSL4 and LPCAT3 promote the formation of phospholipids that are more likely to undergo peroxidation. The balance between ACSL4 and ACSL3/MBOAT activity helps set the threshold for ferroptosis. ACSL4 encourages the incorporation of PUFAs into phospholipids and is linked to a poor prognosis in colorectal cancer.

### 5.4. DHODH and Additional GPX4-Independent Defenses

Another mitochondrial defense is dihydroorotate dehydrogenase (DHODH), which reduces ubiquinone to ubiquinol in the inner mitochondrial membrane. This process helps suppress mitochondrial lipid peroxidation, working alongside mitochondrial GPX4. In tumors with low GPX4, DHODH can help prevent ferroptosis, and blocking DHODH (for example, with brequinar) may make these tumors more sensitive to ferroptosis. Along with FSP1–CoQ10 and GCH1–BH4, DHODH shows that several overlapping, compartment-specific systems often need to be targeted together for effective lipid-peroxidation–dependent cell killing [51].

## 6. Cell Death Dependent on Lipid Peroxidation

The following types of cell death vary in the extent to which they depend on lipid peroxidation. Ferroptosis always requires lipid peroxidation. In contrast, for apoptosis, necrosis, necroptosis, pyroptosis, immunogenic cell death, and lipotoxic death, lipid peroxidation acts more as a modulator or amplifier. It is sometimes permissive or associated, but not always necessary to trigger these forms of cell death.

### 6.1. Ferroptosis

Among non-apoptotic cell death mechanisms, ferroptosis has emerged as a major focus of cancer research in recent years. Although the induction of ferroptosis may promote the elimination of cancer cells, it may also compromise anticancer immunity. Therefore, a comprehensive understanding of this process is essential for developing effective therapeutic strategies. In this process, iron-dependent lipid peroxides accumulate in great amounts in cellular membranes. This may occur as a consequence of the downregulation of lipid ROS detoxification pathways or as a result of direct lipid ROS generation. PUFAs have easily abstractable bis-allylic hydrogens; they are specific substrates of iron-dependent peroxidation, which is a hallmark of ferroptosis [52]. It is worth mentioning that free fatty acids are not inducers of ferroptosis; instead, PLs containing PUFA acyl tails (PUFA-PLs) are the primary drivers of this cell death mechanism [53]. PUFA-PLs can be oxidized by not only enzymatic reactions but also non-enzymatic autoxidation via the Fenton reaction. The enzymes, which take part in enzymatic lipid peroxidation of PUFA-PLs, are arachidonate lipoxygenases (ALOXs) and P450 oxidoreductase POR. The nonheme iron in ALOXs introduces oxygen to PUFAs and PUFA-containing lipids within cell membranes. In non-enzymatic lipid peroxidation of PUFA-PLs, iron is a catalyst. In this pathway, once the initial PLOOHs are generated and are not promptly reduced by GPX4, the Fenton reaction can begin. In this process, PLOOHs interact with ferrous iron to produce alkoxyl and LOO•s, which induce the production of PLOOHs [52]. There are various enzymes that take part in triggering such a complex mechanism as ferroptosis. Acyl-CoA synthetase long-chain family member 4 (ACSL4) plays a crucial role in the activation of long-chain PUFAs by converting them into acyl-coenzyme A derivatives, thereby contributing to membrane phospholipid biosynthesis [50]. Subsequently, ACSL4 has emerged as a promising target in cancer therapy. A 2025 study demonstrated that ACSL4 expression was significantly elevated in colorectal cancer cells and was associated with poor prognosis. Furthermore, ACSL4 suppressed the immune system’s anti-tumor mechanisms by modulating the RIG-I-MAVS-IFN pathway. This shows a promising future direction in CRC immunotherapy [54]. What is more, suppression of ASCL4 might be a useful target in the treatment of other types of tumors, such as breast and prostate cancer. It has been proven that ASCL4 inhibitor PRGL493 sensitized cells to chemotherapy and hormonal therapy [55]. Among all enzymes involved in ferroptosis, the main regulatory role is often attributed to glutathione peroxidase 4 (GPX4), a lipid hydroperoxide scavenger. It is an inhibitor of Ferroptosis, which converts LOOHs into non-toxic lipid alcohols. Recently, there have been advances in understanding its function. GPX4 is thought to prevent ferroptosis by clearing intracellular peroxides and plays an important role in the Xc−/GSH/GPX4 pathway. This axis is a crucial part of the cell antioxidant system. Modulation of GPX4 activity by different inducers or gene knockout has a profound effect on cells, and this potential can be used in anticancer treatment [56]. As an example, elevated GPX4 protein level has been found in HCC cells after triptolide TPL treatment. With the co-administration of TPL and RSL3, a RAS-selective, lethal 3 GPX4 inhibitor, HCC cell apoptosis was promoted; intracellular ROS levels were elevated, and ferroptosis was induced [57]. Another aspect worth highlighting is the role of lipoxygenases (LOX), a specific group of dioxygenase enzymes. They catalyze the double oxidation of both PUFAs and polyunsaturated fatty acyls. They initiate ferroptosis via direct oxidation of arachidonoyl and adrenoyl in phosphatidylethanolamine [56].

### 6.2. Apoptosis

The uncontrolled growth of cells resulting from compromised regulatory mechanisms is considered the beginning of cancerous transformation. The biological mechanism of cancer progression might be associated with dysregulation in cell death programs, including apoptosis—the strictly controlled form of programmed cell death [58]. Apoptosis is a naturally occurring process that eliminates unwanted cells and can be induced by death signals. It plays a crucial role in homogenesis, maintains a stable cell population, and limits the dysfunctional cells. However, any disturbance in that process can cause cancer or autoimmune disease [59]. The mechanism of apoptosis starts with cellular and chromatin condensation. Intense plasma membrane protrusions are followed by nuclear fragmentation called karyorrhexis and a process referred to as “budding” which leads to division of cell fragments known as apoptotic bodies. These structures contain cytoplasmic material with densely packed organelles and may include nuclear fragments or lack them entirely. These bodies are then digested by macrophages, parenchymal cells, or neoplastic cells and subsequently degraded within phagolysosomes [60]. Apoptosis can be initiated by three principal pathways: the extrinsic pathway, the intrinsic pathway, and the perforin/granzyme-mediated pathway. Caspases are important molecules in the transmission of apoptotic signals, initiating numerous biochemical and structural changes. Although the pathways mentioned above can be activated independently, they ultimately converge on the activation of the executioner caspase-3. It mediates the completion of DNA fragmentation via DNA fragmentation factor (DFF) [61]. The extrinsic pathway is triggered when a member of the tumor necrosis factor (TNF) receptor family binds its ligand. This process targets procaspase-8, which is then activated and, in turn, engages caspase-3, leading to the execution phase of apoptosis [62]. The perforin/granzyme pathway is initiated by granzyme B, which targets procaspase-10 or directly activates caspase-3, and granzyme A, which promotes the induction of apoptosis through the inhibitory complex of a DNase [63,64]. The intracellular stressors initiate the intrinsic pathway, which mediates mitochondrial outer membrane permeabilization (MOMP), during which the outer membrane becomes permeable due to activation of specific pro-apoptotic members of the BCL-2 protein family. This process results in the release of pro-apoptotic factors, including cytochrome c (Cyt c). The translocation of cyt c from the mitochondria intermembrane space (IMS) into the cytoplasm ultimately triggers cell death. The promotion of cell death by cyt c begins with the activation of apoptotic protease-activating factor-1 (Apaf-1), forming a caspase-activating complex that subsequently recruits and activates the initiator caspase-9 and the effector caspases-3 and -7. Cyt c is also responsible for the oligomerization of Apaf-1 that creates Apaf-1 apoptosome. This form mobilizes procaspase-9 via caspase recruitment domains (CARDs). After binding, active caspase-9 activates caspase-3, thereby triggering cell death [65,66].

Apoptosis is, in itself, a caspase-driven program that does not require lipid peroxidation; the focus here is therefore restricted to the specific points at which lipid peroxidation intersects apoptotic signaling relevant to cancer therapy, rather than to a general description of the pathway. Lipid peroxidation plays a crucial role in programmed cell death, and its products interact with membrane-localized receptors and transcription factors that activate apoptosis [5]. Lipid peroxidation is initiated by ROS. The mitochondria-specific phospholipid localized in the inner membrane, cardiolipin (CL), is a key component of the intrinsic pathway of cell death, as ROS produced by mitochondria promote CL peroxidation and apoptosis. During apoptosis, cyt c functions similarly to a CL oxygenase, whose activation contributes to the permeabilization of the outer mitochondrial membrane through interactions with Bcl-2 family proteins. The oxidation of CL is a crucial step for the release of pro-apoptotic factors into the cytoplasm. Prior to its release, cyt c dissociates from the inner mitochondrial membrane, where it is normally associated with CL. Endogenous mitochondrial phospholipase A2 slowly converts CL into dilyso-CL. However, the role of mitochondrial phospholipases in CL oxidation remains unclear, since the CL fatty acids attached to cyt c may protect some molecules from oxidation. Ultimately, CL is broken down in lysosomes by lysosomal phospholipase, producing glycerophosphoglycerol and glycerol. While mitochondrial phospholipases can catalyze mono- and di-lysoCL formation, lysosomes appear to be the primary route for removing the remaining fatty acid chains during the early stages of CL degradation [67,68,69,70].

Other products of lipid peroxidation can also promote apoptosis. Essential fatty acids and their metabolites—PUFAs, including gamma-linolenic acid (GLA), AA, EPA, and DHA, are considered to promote programmed cell death. Although the exact mechanism remains unclear, PUFAs have been suggested to promote apoptosis in tumor cells, with increased oxidative stress appearing to be a main mechanism underlying this effect. Due to the presence of multiple double bonds in their structure, PUFAs are highly susceptible to oxidation, which promotes the formation of ROS and enhances lipid peroxidation. As a result, cellular membranes become more vulnerable to oxidative damage. Moreover, tumor cells exposed to PUFAs have been shown to exhibit relatively low levels of endogenous antioxidants, which are essential for neutralizing free radicals and limiting oxidative injury. A reduction in antioxidant capacity weakens the cellular defense system and increases susceptibility to oxidative stress. Studies performed on different tumor cell lines, such as HL-60, SP2/0, and AK-S cells, demonstrated that treatment with PUFAs leads to a significant decrease in intracellular antioxidant content and, at the same time, enhances sensitivity to the cytotoxic effects of fatty acids. Moreover, antioxidant depletion further increases this sensitivity [71].

Lipid peroxidation induced by ROS participates in signaling processes that regulate transcriptional regulators. Aldehyde species generated by lipid peroxidation can react with redox-sensitive signaling proteins and kinases, thereby influencing downstream pathways, including activation of the nuclear factor of kappa light-chain enhancer of B cells (NF-κB). This process modulates NF-κB activity by interfering with the degradation of inhibitory proteins called IκB and altering the phosphorylation of proteins in the pathway, leading to NF-κB activation. Once activated, NF-κB can regulate a number of genes involved in cell survival and death, including anti-apoptotic proteins of the Bcl-2 family. As a result of excessive oxidative stress, the NF-κB pathway can be altered to promote pro-apoptotic effects by inhibiting c-myc gene expression and upregulating stress-response genes such as GADD45. This may lead to activation of the JNK signaling pathway, ultimately causing apoptosis. This pathway may explain how ROS-induced lipid peroxidation can alter the activity of transcriptional regulator networks involved in apoptosis [5,72,73].

### 6.3. Necrosis

As with apoptosis, this section is condensed to emphasize the lipid peroxidation–specific contribution to necrotic membrane failure rather than recapitulate general necrosis biology. Necrosis is permanent cell injury resulting from pathological mechanisms, including the following forms of cell death. It is an uncontrolled form of cell death in which the cell and its organelles swell, the plasma membrane breaks, and the cell eventually ruptures, releasing its contents into the surrounding tissue and causing damage. This process is triggered by extracellular stimuli and results in inflammatory responses due to the extracellular release of heat shock proteins, uric acid, ATP, DNA, and nuclear proteins, which promote inflammasome activation. Necrotic cell death was initially considered a consequence of cellular injury, leading to irreversible failure of bioenergetic homeostasis; however, recent studies have shown that necrosis can also occur in a regulated manner, such as in necroptosis or ferroptosis, where specific signaling pathways and molecular mediators control the cell death process. The most common stimuli that can induce necrosis are hypoxia, trauma, chemical substances, biological agents such as bacteria, viruses, or fungi, and autoimmune responses [74,75].

The necrotic cell shows organelle swelling, including the ER and mitochondria, followed by loss of plasma membrane integrity and ultimate cellular disintegration. Cell injury is associated with a decline in intracellular ATP levels due to impaired production. Under these circumstances, the ATP-dependent sodium-potassium pump (Na^+^-K^+^-ATPase) is impaired due to ATP deficiency, disrupting ion homeostasis and leading to accumulation of sodium, chloride, and water in the cell, resulting in cell swelling. The Na^+^ overloading, which leads to energy failure and cellular dysfunction, results in cell swelling and necrosis [76,77]. Moreover, Na^+^ accumulation activates nonselective Ca^2+^ channels, thereby increasing intracellular calcium. High cytosolic calcium, together with oxidative stress, damages mitochondria and activates enzymes such as phospholipases and proteases, promoting the degradation of membranes and proteins [78,79].

Phospholipases A2 (PLA2) play a crucial role in necrosis by mediating the release of arachidonic acid (AA) from membrane phospholipids (PLs). To access their substrates and release AA, cytosolic PLA2 (cPLA2) needs to be translocated to cellular membranes, and this process requires Ca^2+^. The release of AA by PLA2 is crucial for ROS and lipoxygenases (LOXs), which catalyze the conversion of PUFAs, including AA, into LOOHs. This mechanism promotes lipid peroxidation that weakens membranes and leads to necrotic cell death. LOOHs can also be formed through non-enzymatic lipid peroxidation. Lipid peroxides can also be generated by mitochondrial dysfunction, as it produces hydroxyl radicals that attack unsaturated fatty acids. Accumulation of these LOOHs can disrupt organelle and plasma membranes, resulting in membrane rupture and necrotic cell death [80]. Moreover, elevated Ca^2+^ levels and ROS can trigger the opening of the mitochondrial permeability transition pore (mPTP), which is regulated by cyclophilin D (CypD). This increases mitochondrial membrane permeability, leading to loss of the proton gradient and ATP production, ultimately resulting in mitochondrial swelling, outer membrane rupture, and concurrent damage to cellular membranes and organelles [78]. Thus, in necrosis lipid peroxidation acts mainly as one membrane-destabilizing input among several, not as an obligatory trigger.

### 6.4. Necroptosis

Necroptosis is a form of regulated cell death that depends on RIPK3 and the pseudokinase MLKL. It can be triggered by death receptors such as TNFR1 and FAS, or by pattern recognition receptors (PRRs) like TLR3, TLR4, and ZBP1 [81]. Compared with apoptosis, necroptosis is a caspase-independent process. In cells lacking caspase-8, TNFα induces the formation of RIPK1/RIPK3 complexes called the necrosome. RIPK3 forms oligomers and activates MLKL, which changes shape and exposes its “killer” 4HB domain. This process induces oligomerization and promotes MLKL transport to the plasma membrane. Once there, MLKL inserts into the membrane, forming pores and causing necroptotic cell death. Lipids like phosphoinositides and highly phosphorylated inositol phosphates help MLKL attach to the membrane and release its auto-inhibition. Very-long-chain fatty acids can enhance necroptosis by affecting membrane organization, while lipid rafts may facilitate the removal of phosphorylated MLKL during membrane repair [82,83,84].

Recent studies show that lipid peroxidation can modulate, but is not strictly essential for, necroptosis: necroptosis is canonically defined by RIPK1/RIPK3/MLKL signaling and can proceed without overt lipid peroxidation, although lipid ROS can promote it in specific contexts. PUFAs or extensive accumulation of lipid peroxides, such as 4-HNE, as well as depletion of protective enzymes like GPX4, promote RIPK3 activation and MLKL-mediated necroptotic cell death. A study conducted in mice demonstrated that GPX4 suppresses necroptosis in erythroid cells. This process demonstrated that GPX4 prevented cell death by regulating cellular redox balance, and its absence induced programmed necrosis, including necroptosis [85]. The common stress inducer sodium iodate (NaIO_3_) induces necroptosis in retinal pigment epithelial (RPE) cells. Nec-1, which is an RIPK1 and necrosome inhibitor, while NSA is an MLKL inhibitor. Potential inhibitors of necroptosis, such as Nec-1 and necrosulfonamide (NSA), could have therapeutic potential for diseases, including RPE cell death; further research is needed. Mitochondrial dysfunction, especially the overactivation of respiratory chain complex I, increases cellular lipid ROS, thereby increasing redox-active iron. This process can induce Fenton reactions that further contribute to necrosome formation. The following cell lysis releases intracellular contents, including danger-associated molecular patterns (DAMPs), which stimulate inflammatory responses. Lipid peroxidation-induced cell death can lead to tissue damage and may, in turn, promote carcinogenesis. Under conditions of tissue injury or chronic oxidative stress, this process can contribute to inflammatory toxicity, whereas in cancer it can generate an immune response to dying cells through increased immune system activity, resulting from greater antigen presentation, and thereby stimulate the anti-tumor immune reaction. Pharmacological or genetic interventions, such as necrostatin-1, GPX4 overexpression, antioxidants, or iron chelators, demonstrate that controlling lipid ROS can influence both the extent of necroptosis and its downstream inflammatory or immunogenic effects. The evidence indicates a strong connection between lipid peroxidation and necroptosis, as they may promote either harmful inflammatory responses or beneficial anti-tumor responses, depending on the context [2,86,87].

### 6.5. Pyroptosis

Pyroptosis represents a unique form of programmed cell death. It is initiated by microbial infection or danger signals. This process is accompanied by inflammasome activation and the secretion of pro-inflammatory cytokines. Although the mechanism of pyroptosis is not yet fully understood, numerous studies indicate its key involvement in many human diseases, including malignant cancers. It has been suggested that the induction of pyroptosis in tumor cells may inhibit tumor growth [88,89,90].

Inflammasomes, which induce pyroptosis, are cytoplasmic, multiprotein signaling complexes of the innate immune system. Their activation may occur by either the canonical or noncanonical pathway, depending on the type of initiating stimulus [88,89,91]. Pyroptosis is driven by inflammasome assembly and caspase activity, not by lipid peroxidation: in the canonical pathway, cytosolic PRRs recognize PAMPs/DAMPs and, with ASC, activate caspase-1, which cleaves gasdermin D (GSDMD) and the IL-1β/IL-18 precursors; in the non-canonical pathway, human caspase-4/-5 (caspase-11 in mice) directly senses intracellular LPS. These caspases are activated through the direct binding of intracellular lipopolysaccharide or oxidized lipids by the N-terminal caspase recruitment domain (CARD) [89,90,92,93,94]. Lipid peroxidation is best regarded as permissive or amplifying—oxidized lipids can contribute to non-canonical caspase activation and lipid-derived signals can potentiate inflammasome activity rather than as the central trigger of pyroptotic signaling [91,92,95,96].

Caspases participating in both pathways are responsible for the cleavage and activation of GSDMD, a member of the gasdermin (GSDM) protein family, resulting in the formation of a C-terminal fragment (C-GSDMD) and an N-terminal fragment (N-GSDMD) [88,92,93]. N-GSDMD undergoes oligomerization and disrupts the plasma membrane by forming nonselective pores. This leads to cell swelling, cytoplasmic leakage, and ultimately pyroptotic cell death. At the same time, mature IL-1β and IL-18 are released through these pores. This mechanism occurs in macrophages as well as in other immune cells, such as neutrophils [88,92,93,97]. Cytokines released during pyroptosis act as effector molecules that influence antitumor immunity in two different ways, depending on the TME [88,95,98]. Chronic inflammation contributes to the formation of the TME, in which hypoxic regions are present, and macrophages readily accumulate. Hypoxia increases GSDMC expression, which, when activated by caspase-8, induces pyroptosis, thereby promoting tumor progression and suppressing antitumor immune responses. Conversely, acute inflammation leads to macrophage-mediated phagocytosis of tumor cells and increases the number and activity of NK cells and CD^8+^ T lymphocytes within the tumor. CD^8+^ T lymphocytes release granzymes that activate GSDMB, thereby promoting pyroptosis. This process also enhances antitumor activity by releasing the HMGB1 protein [88,93,95].

Antitumour pyroptosis has been shown in GSDME-high colorectal cells (TNFα/CHX→BAK/BAX/MOMP), photoactivated GSDME-mediated pyroptosis in breast cancer, GSDMD/caspase-4 pyroptosis by α-NETA in ovarian cancer [95,99,100]. GSDME can also convert caspase-3-mediated apoptosis into pyroptosis after treatment with chemotherapy drugs such as 5-fluorouracil in gastric cancer cells [89,101]. Other studies also indicate the antitumor role of pyroptosis in liver cancer, bladder cancer, and lung adenocarcinoma [88,93,102,103].

### 6.6. Cuproptosis

Cuproptosis represents a non-apoptotic pathway of cell death in which copper, as a key element, binds to lipoylated components of the tricarboxylic acid (TCA) cycle. Although cuproptosis is mechanistically distinct from lipid peroxidation—its proximal cause is copper-dependent aggregation of lipoylated TCA proteins and proteotoxic stress rather than PUFA oxidation—it is included here because it intersects the same redox and mitochondrial biology: copper ionophores increase ROS, and copper- and iron-dependent death can be co-induced and can cooperate with lipid peroxidation. Copper enters the cell through ionophores. Cells dependent on mitochondrial respiration exhibit nearly 1000-fold greater sensitivity to copper ionophores compared with cells that primarily rely on glycolysis. Elesclomol is a well-characterized copper ionophore whose molecular target is ferredoxin 1 (FDX1), a reductase that reduces Cu^2+^ to Cu^+^, thereby promoting ROS generation [94,102,103]. Copper binds to lipoylated TCA cycle components, such as dihydrolipoamide S-acetyltransferase (DLAT), a mitochondrial protein whose lipoylation is enhanced by FDX1. Copper also promotes the aggregation of lipoylated proteins and the destabilization of iron–sulfur cluster–containing proteins, leading to proteotoxic stress and ultimately cell death [104,105,106]. It appears to play a pivotal role in tumor growth and metastasis, as malignant cells demonstrate an increased demand for this metal [3]. Moreover, it may induce cancer cell death through apoptosis induction or ROS accumulation, making it a potential therapeutic target [3]. The intracellular copper-dependent protein MEMO1 is oncogenic properties and is required for breast cancer cell migration and invasion in vitro, as well as for lung metastasis in vivo. Inhibition of the copper-binding site of MEMO1 suppresses metastatic dissemination of cancer cells [107]. In patients with HCC, resistance to cuproptosis has been associated with reduced FDX1 expression [108]. In ARID1A-deficient HCC, tumor cells become highly dependent on the TCA cycle, suggesting that copper modulation may represent a potential therapeutic strategy in this molecular subtype [109]. Cuproptosis inhibited by arecoline may increase the survival of cancer-associated fibroblasts (CAFs) in patients with oral squamous cell carcinoma [110]. Altered copper levels have also been observed in other malignancies, including leukemia, pancreatic cancer, thyroid cancer, colorectal cancer, and prostate cancer [94]. In a study by Li et al., the synergistic combination of cuproptosis and ferroptosis using nanocarrier platforms was shown to enhance anticancer efficacy. A core–shell nanoparticle system, CuP/Er, composed of copper and erastin, simultaneously induces both forms of cell death, resulting in a more potent combined antitumor response. This effect is mediated by suppression of aerobic glycolysis and inhibition of the TCA cycle, increased lipid peroxidation, and irreversible mitochondrial damage. This strategy effectively inhibited the growth of breast and colorectal tumors in murine models [111].

### 6.7. Immunogenic Cell Death

Immunogenic cell death (ICD) is a form of regulated cell death that increases the immunogenicity of tumor tissue and, therefore, represents a promising strategy for anticancer therapy. It can be induced by chemotherapy, radiotherapy, and other approaches, including photodynamic therapy, physicochemical therapies, and oncolytic viruses. ICD is based on the release of DAMPs by dying tumor cells and/or their exposure on the cell surface, which activates an immune response within the TME [112,113,114]. DAMPs can be divided into constitutive DAMPs (cDAMPs), which are continuously present, endogenous immunostimulatory molecules released by dying cells, and inducible DAMPs (iDAMPs), which are generated during cell death. The most described DAMPs include calreticulin (CRT), heat shock proteins (HSP70 and HSP90), ATP, HMGB1, and cytokines such as interferons and interleukins. DAMPs modulate phagocytosis and antigen presentation through interactions with PRRs, enabling their recognition by NK cells and dendritic cells, which subsequently activate the immune response. In addition, DAMPs contribute to the formation of a pro-inflammatory TME [112,113,114,115,116]. The ability of tumor cells to undergo ICD can be modulated by metabolic alterations, including oxidative stress, enhanced glycolysis, and changes in lipid metabolism. Lipid peroxidation and ferroptosis are key processes that regulate ICD. During these processes, lipid-derived DAMPs are generated, including lipid peroxides and the lipid aldehyde 4-HNE, which, together with OxPL, accelerate dendritic cell maturation and increase the recruitment of CD^8+^ T lymphocytes to the tumor site [113,117].

ICD exhibits synergistic activity with therapeutic approaches such as chemotherapy and radiotherapy, and its antitumor efficacy has been confirmed in both in vitro and in vivo models. ICD inducers include anticancer drugs such as anthracyclines, taxanes, cyclophosphamide, bortezomib, crizotinib, and oxaliplatin, which trigger a CD^8+^ T-cell-dependent immune response leading to tumor cell death [112,113,118]. This mechanism may have significant implications for the treatment of colorectal cancer, pancreatic cancer, gastric cancer, and hepatocellular carcinomas [119]. A growing body of clinical data suggests that irradiated tumors can be converted from “cold” tumors into “hot” lesions through more frequent stimulation of ICD. This increases the sensitivity of tumors that are otherwise unresponsive to immunotherapy, supporting the rationale for combining immunotherapy with radiotherapy in clinical practice. Currently used approaches include photon radiotherapy, proton therapy, carbon-ion therapy, and boron neutron capture therapy [118,119].

### 6.8. Lipid-Induced Cell Death

Maintaining lipid homeostasis is necessary for cellular viability and metabolic stability. Lipids work not only as structural components of biological membranes but also as energy substrates and signaling molecules regulating numerous cellular processes. Disturbances in lipid metabolism could lead to excessive lipid accumulation in non-adipose tissues. Excessive intracellular lipid accumulation, known as lipotoxicity, contributes to metabolic dysfunction and cellular injury. Although the term ‘lipidoptosis’ is not formally recognized within the established classification of regulated cell death mechanisms, it may serve as a useful conceptual framework for describing cell death driven by pathological lipid accumulation and organelle dysfunction. In this context, lipid-induced cell death represents a form of metabolic failure in which molecules that normally fulfill structural and metabolic roles become cytotoxic and disrupt cellular homeostasis. Lipotoxicity has been implicated in the pathogenesis of numerous metabolic and degenerative disorders, including obesity, type 2 diabetes, cardiovascular disease, and metabolic-associated fatty liver disease (MASLD) [120,121]. Excess SFA, like palmitic acid, can trigger multiple stress pathways. These include oxidative stress, inflammation, mitochondrial dysfunction, and endoplasmic reticulum stress. Together, they contribute to cellular injury and disease progression [122].

Mechanistically, lipid-induced cytotoxicity is a multifactorial process involving interconnected pathways, including mitochondrial dysfunction, endoplasmic reticulum (ER) stress, oxidative stress, and changes in membrane lipid composition [123,124]. Cells initially buffer lipid overload by storing fatty acids as neutral triglycerides within LD [125]. These dynamic organelles are composed of a neutral lipid core surrounded by a phospholipid monolayer. The cellular LD content increases in response to NRF2 stabilization under lipotoxic stress. NRF2 increases the expression of G0S2, a protein that inhibits the ATGL enzyme, which is responsible for the degradation of triacylglycerol and the release of fatty acids from LDs. Stabilization of NRF2 therefore increases the cellular LD content, while inhibition of ATGL partially eliminates the effect of NRF2 deletion on ferroptosis. Stress-induced stabilization of NRF2 allows for temporary protection of the cell against lipotoxicity by sequestering fatty acids in the LDs [126]. When this storage capacity is exceeded, toxic lipid species accumulate in membranes and organelles, initiating stress responses that progressively compromise cell structure integrity [125]. Under such conditions, lipolysis is chronically elevated, leading to cell damage, which is likely related to increased levels of free fatty acids in the cytosol [127].

Interfering with LD metabolism in cancer cells appears to be an interesting therapeutic target. Inhibiting LD biosynthesis may disrupt metabolic pathways crucial for cancer cell survival and may increase lipotoxicity, thereby blocking important survival mechanisms. Potential molecular targets include adipose triglyceride lipase (ATGL), lysophosphatidylcholine acyltransferase 2 (LPCAT2), and diacylglycerol acyltransferase 1 (DGAT1). It should be noted, however, that lipid metabolic pathways can have both inhibitory and pro-tumor growth effects. For example, increased ATGL expression in breast cancer is correlated with increased fatty acid oxidation, which increases the ability of cancer cells to metastasize. In prostate cancer, tumor activity is negatively correlated with ATGL expression [127,128].

#### 6.8.1. Organelle Stress and Metabolic Dysfunction

As lipid accumulation advances, intracellular organelles become more susceptible to lipotoxic stress. Mitochondria and the endoplasmic reticulum (ER) are particularly vulnerable due to their central roles in cellular energy. metabolism and lipid biosynthesis. Lipid accumulation can disrupt ER-mitochondrial crosstalk, thereby promoting mitochondrial remodeling, oxidative stress, and apoptosis [123]. The sites of contact between the ER and mitochondria, known as mitochondrial-associated membranes (MAMs), facilitate the exchange of lipids, numerous ions, and metabolites. Furthermore, they allow the cell to regulate its bioenergetics and participate in essential processes regulating cell fate in response to stress factors [128,129]. Sphingolipids, including ceramide, modulate mitochondrial function. Ceramide, like other sphingolipids, is biosynthesized in the ER and transported to mitochondria through mitochondria-associated membranes (MAMs). Their transport via the ceramide transport protein (CERT) further enhances mitochondrial stress and may trigger apoptotic signaling. Its accumulation impairs mitochondrial respiration and promotes mitochondrial dysfunction. Medium-chain ceramides are responsible for the formation of channels in the outer mitochondrial membrane (OMM), which increases membrane permeability and allows the release of intermembrane proteins with proapoptotic properties [130]. Similarly, lipotoxic stress activates ER stress signaling pathways and the unfolded protein response, which may initially promote adaptation but, in the end, contribute to cellular dysfunction and apoptosis if chronically activated [123].

#### 6.8.2. Membrane Destabilization

Alterations in membrane lipid composition are another essential component of lipid-induced cytotoxicity. Shifts in phospholipid and sphingolipid balance can modify membrane fluidity, disrupt signaling complexes, and impair organelle function [120]. Membrane disturbances may also promote excessive generation of ROS, consequently amplifying oxidative stress and cellular injury [122]. Ceramides play a key role in modifying membrane properties. Once incorporated into membranes, they increase the stiffness of PLs in membranes, induce phase separation and domain formation in lipid bilayers. They possess a distinct internal negative curvature that facilitates the formation of hexagonal phases, increases membrane permeability to large substances, promotes transmembrane lipid movement, and are highly hydrophobic, preventing them from suspending in an aqueous environment [131].

#### 6.8.3. Terminal Cellular Failure

Excessive intracellular lipid accumulation disrupts cellular homeostasis through multiple interconnected mechanisms, including lipotoxicity, formation of toxic lipid intermediates, mitochondrial dysfunction, ER stress, oxidative stress, and membrane destabilization. Together, these processes contribute to organelle failure, metabolic imbalance, and cellular injury. When lipotoxic stress exceeds the cell’s adaptive capacity, multiple cell death pathways may be activated. Lipotoxicity has been shown to induce apoptosis, inflammatory responses, and other forms of regulated cell death, thereby contributing to tissue damage and disease progression [120,121]. The major cellular mechanisms involved in lipid-induced cell damage are summarized in Table 1.

#### 6.8.4. Stages of Lipid-Induced Cellular Damage

Lipid-induced cellular injury develops through a progressive sequence of metabolic and structural disturbances. The initial stage is characterized by disruption of the balance among lipid uptake, synthesis, storage, and degradation. Elevated circulating fatty acids accumulate in tissues not specialized for lipid storage, resulting in cellular dysfunction and metabolic stress [121]. High concentrations of SFA, particularly palmitic acid, are recognized as potent inducers of lipotoxicity. Experimental studies prove that palmitate exposure triggers oxidative stress, ER stress, mitochondrial dysfunction, and apoptotic cell death [122]. When intracellular lipid levels exceed the capacity for metabolic detoxification or storage, bioactive lipid intermediates such as ceramides and diacylglycerols accumulate. Ceramides have been recognized as key mediators linking lipid overload with metabolic disease and cellular dysfunction [120]. Ceramide plays a key role in vascular dysfunction in diet-induced obesity. At the cellular level, this mechanism involves dephosphorylation of endothelial nitric oxide synthase (eNOS) by protein phosphatase 2A (PP2A). It has been demonstrated that in endothelial cells, ceramide binds to PP2A inhibitor 2 (I2PP2A) in the cytosol, thereby impeding I2PP2A binding to PP2A and, consequently, allowing PP2A translocation to the plasma membrane. This increases the association between PP2A and eNOS at the plasma membrane, promoting the dissociation of the Akt-Hsp90-eNOS complex, which plays a key role in eNOS phosphorylation and activation. Inhibition of PP2A maintained normal blood pressure in obese mice [132]. In insulin resistance, ceramides act by antagonizing insulin. This involves inhibiting phosphatidylinositol-3 kinase (PI3K) signaling and blocking the activation of the anabolic enzyme Akt/PKB, thereby disrupting glucose uptake by tissues [133].

#### 6.8.5. Mitochondrial Response to Lipid Stress

Mitochondria are central to the cellular response to lipid overload. As the primary site of fatty acid oxidation, mitochondria experience a substantial metabolic burden when exposed to excess lipids [134]. Elevated levels of SFA stimulate β-oxidation, increasing electron flow through the mitochondrial respiratory chain and promoting mitochondrial stress responses [135]. This metabolic pathway intensification increases the production of ROS, which causes oxidative damage to mitochondrial proteins, lipids, and mitochondrial DNA [136]. Oxidative stress can also lead to depolarization of the mitochondrial membrane potential, a critical event associated with mitochondrial dysfunction and activation of cell death pathways [137].

An important process in this context is mitochondrial permeability transition (mPT). This phenomenon involves an increase in the permeability of the inner mitochondrial membrane, allowing the diffusion of solutes with molecular masses of up to 1.5 kDa. This process is Ca^2+^-dependent and facilitated by CypD. Solute transport occurs through a nonselective channel called the mPTP. Short-term opening of the mPTP may play a physiological role, as it can facilitate rapid Ca^2+^ efflux or alter mitochondrial bioenergetics. Persistent opening of the mPTP causes mitochondrial swelling and disrupts the outer mitochondrial membrane, leading to the release of pro-apoptotic factors and activation of apoptosis [138]. Pro-apoptotic proteins such as BAX and BNIP3 are activated during lipotoxic stress. BAX promotes mitochondrial outer MOMP, resulting in the release of pro-apoptotic factors and activation of caspase-dependent apoptosis [139]. BNIP3, induced under cellular stress conditions, contributes to mitochondrial dysfunction and stimulates mitophagy and mitochondrial fragmentation as part of stress-response pathways [140]. Moreover, lipotoxic stress promotes mitochondrial fragmentation primarily mediated by dynamin-related protein 1 (DRP1) [141]. Excessive mitochondrial fission is associated with impaired respiratory capacity and elevated susceptibility to apoptotic signaling, especially under lipotoxic conditions [142].

Autophagy also plays an important role in the mitochondrial response to lipid-related stress. In the context of lipotoxicity, it helps mitigate the harmful effects of lipid accumulation. Autophagy also influences processes such as lipogenesis, lipolysis, and fatty acid oxidation, allowing cells to adapt to environmental changes and maintain lipid homeostasis [143]. Mitophagy, a selective form of autophagy, normally functions as a quality-control mechanism that removes damaged mitochondria, protecting cells from excessive oxidative stress. Chronic lipid overload, however, can overwhelm mitochondrial quality-control systems, resulting in the accumulation of dysfunctional mitochondria that generate excessive ROS and exacerbate cellular damage [144,145]. Different types of fatty acids may differentially regulate autophagic pathways. Determining how saturated and unsaturated fatty acids affect the autophagy process could lead to improved pharmacological strategies and dietary recommendations aimed at limiting the effects of lipotoxicity [143] (See Table 2).

#### 6.8.6. Endoplasmic Reticulum Stress Pathway

Accumulation of SFA alters the lipid composition and physicochemical properties of the ER membrane, thereby disturbing protein folding processes and promoting ER stress. Changes in ER membrane lipid composition, often referred to as lipid bilayer stress, have been shown to directly activate sensors of the unfolded protein response (UPR) [146]. Membrane aberrancies can be sensed directly by ER stress transducers such as IRE1, which respond to alterations in membrane properties [147]. In response to these disturbances, cells initiate the UPR signaling network, which works to restore ER homeostasis by enhancing protein folding capacity, attenuating protein synthesis, and promoting quality-control mechanisms [148]. The UPR is mediated by three principal signaling branches, including protein kinase RNA-like ER kinase (PERK), activating transcription factor 6 (ATF6), and inositol-requiring enzyme 1α (IRE1α) [147]. Activation of these pathways reduces the burden of unfolded proteins in the ER, increases molecular chaperone expression, and promotes protein degradation through ER-associated degradation [149]. PERK activation results in phosphorylation of the eukaryotic initiation factor eIF2α, which transiently attenuates global protein translation and reduces the influx of newly synthesized proteins into the ER. Despite this general inhibition of translation, phosphorylation of eIF2α promotes selective translation of activating transcription factor 4 (ATF4), which regulates genes involved in cellular stress adaptation and metabolic regulation [150]. ATF6 functions as a transcription factor that, following ER stress, translocates to the Golgi apparatus, where it receives proteolytic activation [151]. The cleaved form of ATF6 subsequently induces the expression of ER chaperones, such as GRP78/BiP, which enable protein folding and help restore ER homeostasis [150,151]. The third branch of the unfolded protein response comprises the ER-resident sensor IRE1α, which possesses both kinase and endoribonuclease activities [152]. Upon activation, IRE1α catalyzes the unconventional splicing of XBP1 mRNA, generating an active transcription factor that regulates UPR target genes involved in protein folding and ER-associated degradation [153]. Although the UPR initially functions as an adaptive mechanism that promotes cell survival, prolonged or severe ER stress can shift the cellular response toward apoptotic signaling pathways [150,154]. Chronic ER stress has been implicated in metabolic disorders associated with lipid accumulation, including obesity, insulin resistance, and non-alcoholic fatty liver disease [155]. Table 3 presents key signaling pathways of the unfolded protein response (UPR).

## 7. Dual Pro- and Anti-Tumor Roles of Lipid Peroxidation

Lipid peroxidation does not act uniformly as an anticancer mechanism. At high levels, it induces tumor cell death, particularly through ferroptosis, and can promote immunogenic cell death, supporting its therapeutic potential. However, chronic, sub-lethal exposure can be pro-tumorigenic: 4-HNE– and MDA–DNA adducts are mutagenic; ongoing oxidative signaling via NRF2, NF-κB, and related pathways can enhance proliferation, survival, and stemness; and inflammation driven by lipid peroxidation can create an immunosuppressive, pro-metastatic microenvironment [156]. The therapeutic objective is not simply to increase lipid peroxidation, but to drive tumor cells acutely past the lethal threshold while minimizing chronic, sub-lethal oxidative damage to normal tissue. This duality is a key regulation for LPO-based therapies and informs the translational challenges discussed below.

## 8. Translational Challenges and Opportunities

Most strategies to modulate lipid peroxidation still remain at the preclinical stage, and several translational challenges must be addressed. Regarding tumor selectivity and normal-tissue toxicity, iron loading and GPX4/SLC7A11 inhibition can harm normal tissues such as the kidney, intestine, and neurons, so defining a therapeutic window is essential. Given their pharmacology and delivery, many ferroptosis inducers, including RSL3 and erastin, have poor bioavailability and lack clinical validation, prompting the development of nanoparticle and prodrug delivery systems, such as RSL3- or PUFA-loaded liposomes and CuP/Er particles. The most challenging are resistance and combination strategies, because GPX4, FSP1–CoQ10, DHODH, SLC7A11, NRF2, ALDH, and PON2 offer redundant protection, effective treatment will likely require rational drug combinations, such as GPX4 plus FSP1, SCD1 inhibition with a ferroptosis inducer, or NRF2 inhibition to restore sensitivity [51,157,158]. However, the most crucial are biomarkers; lineage- and state-specific dependence necessitates patient selection markers, including ACSL4, SCD1, FSP1, GPX4, and NRF2/KEAP1 status [159]. Clinical translation is in its early stages, but combinations of ferroptosis-relevant agents with chemotherapy are under investigation (e.g., NCT03247088) [160]. Table 4 summarizes key pharmacological agents, their main targets, and current development status.

## 9. Conclusions

This review summarizes the physiological and pathophysiological roles of lipid peroxidation in the body and its impact on the efficacy of anticancer drugs. MDA and 4-HNE adducts play major roles in numerous metabolic pathways and may participate in secondary deleterious reactions. However, MDA is considered a major mutagenic lipid peroxidation product, whereas 4-HNE is regarded as one of the most cytotoxic. Notably, lipid peroxidation can modulate cellular signal transduction by regulating proliferation, differentiation, and cell death. Tumor cells can remodel lipid metabolism either actively or passively, thereby becoming resistant or susceptible to certain pharmacological agents. Additionally, one of the recently recognized lipid peroxidation-related processes is iron-dependent ferroptosis, a form of regulated cell death that plays an important role in anticancer treatment. Uncontrolled cell growth reflects a disruption of regulatory mechanisms and is considered an early event in carcinogenesis. Moreover, lipid peroxidation products may interact with membrane receptors and transcription factors that mediate apoptosis. Several forms of regulated cell death can occur simultaneously in cells, including necrosis, cuproptosis, immunogenic cell death (ICD), and lipid-induced cell death. These processes contribute to the regulation of lipid homeostasis, which is essential for cellular viability and stability. The important role of mitochondria in regulating oxidative stress responses in the body should also not be overlooked.

To summarize, the process of lipid peroxidation is not yet fully understood. However, several clear conclusions come from the current evidence. First, among controlled cell-death processes, only ferroptosis fully depends on lipid peroxidation, whereas the others are influenced to varying degrees depending on the situation. This difference should guide the choice of death types targeted in specific tumors. Second, the most useful targets for treatment include GPX4, the related FSP1–CoQ10/DHODH systems (which might work better if blocked together), the SREBP1–SCD1/MUFA pathway (which could be combined with mTOR inhibition), SLC7A11, cancer-causing NRF2, and the antioxidant enzymes ALDH and PON2. Third, the biggest gaps in knowledge involve finding the right treatment window that uses both the cancer-promoting and cancer-fighting effects of lipid peroxidation, developing markers that predict dependence based on cell type and state, improving how ferroptosis inducers are delivered and targeted, and producing strong clinical evidence beyond early studies like NCT03247088. Filling these gaps, rather than just listing more connections, is key to translating lipid peroxidation biology into effective and lasting cancer treatments.

## Figures and Tables

**Table 1:** Major mechanisms underlying lipid-induced cellular damage and their biological consequences.

Mechanism	Key Cellular Events	Biological Consequences	Relevance to Lipid-Induced Cell Damage	References
Lipid overload/lipotoxicity	Excessive uptake and intracellular accumulation of fatty acids in non-adipose tissues	Metabolic imbalance, activation of stress pathways, impaired cellular homeostasis	Initiates metabolic disturbance leading to organelle dysfunction and cell damage	[119,120]
Ceramide and lipid intermediate accumulation	Increased levels of bioactive lipids, including ceramides and diacylglycerols	Disrupted signaling, mitochondrial dysfunction, and insulin resistance	Links altered lipid metabolism to stress signaling and apoptosis susceptibility	[119]
Mitochondrial dysfunction	Enhanced β-oxidation and mitochondrial stress responses	ROS generation, mitochondrial depolarization, impaired ATP production, apoptosis	Central driver of oxidative damage and energy failure	[131,132,133]
Endoplasmic Reticulum Stress	Disruption of protein folding due to altered ER membrane composition	Activation of unfolded protein response (UPR), proteostasis imbalance	Reflects failure of adaptive stress responses and promotes cell death pathways	[133,134]
Oxidative stress	Increased production of reactive oxygen species (ROS)	Damage to proteins, lipids, and mitochondrial DNA	Amplifies lipid-induced damage and promotes irreversible cellular injury	[135]
Membrane destabilization	Altered phospholipid and sphingolipid composition	Impaired membrane integrity and signaling, organelle dysfunction	Disrupts membrane structure and cellular compartmentalization	[119]

**Table 2:** Mitochondrial alterations associated with lipid overload.

Mechanism	Key Molecular Mediators	Cellular Consequences	Relevance to Lipid-Induced Cell Damage	References
Enhanced fatty acid β-oxidation	Mitochondrial respiratory chain enzymes	Increased electron flux and mitochondrial stress	Promotes electron leakage and predisposes mitochondria to oxidative injury	[13,14]
Reactive oxygen species (ROS) generation	Mitochondrial ROS	Oxidative damage to mitochondrial proteins, lipids, and DNA	Amplifies mitochondrial dysfunction and contributes to irreversible cellular injury	[135]
Mitochondrial membrane depolarization	Loss of mitochondrial membrane potential (ΔΨm)	Activation of mitochondrial cell death pathways	Indicates bioenergetic failure and increased susceptibility to apoptosis	[138]
Mitochondrial fragmentation	DRP1-mediated mitochondrial fission	Impaired mitochondrial respiration and increased apoptotic susceptibility	Reflects structural remodeling associated with mitochondrial stress and dysfunction	[142,143]
Activation of pro-apoptotic proteins	BAX, BNIP3	Mitochondrial outer membrane permeabilization (MOMP), mitophagy	Links mitochondrial injury to apoptosis and selective organelle turnover	[140,141]
Mitophagy	Autophagy machinery	Removal of damaged mitochondria	Represents an adaptive quality-control mechanism that may limit mitochondrial damage	[146]

**Table 3:** Major signaling branches of the unfolded protein response (UPR) and their cellular consequences.

UPR Branch	Key Molecular Components	Core Mechanism	Cellular Consequences	Functional Relevance in ER Stress	References
PERK pathway	PERK, eIF2α, ATF4	PERK phosphorylates eIF2α, attenuating global protein translation while promoting ATF4 translation	Reduced ER protein load, stress adaptation	Limits protein overload and promotes adaptive stress responses under ER stress	[150,151]
ATF6 pathway	ATF6, GRP78/BiP	ATF6 translocates to the Golgi apparatus, where it undergoes proteolytic activation	Increased expression of ER chaperones and folding-related proteins	Enhances ER protein-folding capacity and supports restoration of proteostasis	[150,151]
IRE1α pathway	IRE1α, XBP1	IRE1α mediates unconventional splicing of XBP1 mRNA	Upregulation of genes involved in protein folding, lipid biosynthesis, and ER-associated degradation (ERAD)	Coordinates adaptive remodeling of ER function and quality-control pathways	[152,153]

**Table 4:** Representative pharmacological agents that modulate lipid peroxidation, their principal molecular targets and effects, and indicative clinical/preclinical status.

Agent	Principal Target	Effect on LPO Axis	Status (Indicative)
Sorafenib	Multikinase (RAF/VEGFR/PDGFR); indirectly system xc−	Promotes lipid ROS via xc− inhibition (context-dependent)	Approved (HCC, RCC); ferroptosis role investigational
Cisplatin	DNA cross-links; mitochondrial ROS; GPX4 (context)	Secondary lipid peroxidation downstream of oxidative stress	Approved (many solid tumors)
Olaparib	PARP; p53-dependent SLC7A11 down-regulation	Sensitizes to ferroptosis; enhances inducers	Approved (BRCA/HRD cancers)
Erastin/IKE	SLC7A11 (system xc−)	Depletes GSH → ferroptosis	Preclinical (tool/IKE optimized)
RSL3/ML210/ML162	GPX4	Direct GPX4 inhibition → lipid-peroxide accumulation	Preclinical tool compounds
iFSP1/FSeIII	FSP1 (AIFM2)	Blocks CoQ10-based radical trapping; synergy with GPX4 inhibition	Preclinical
Brequinar	DHODH	Restores mitochondrial lipid peroxidation in GPX4-low tumors	Repurposing/preclinical for ferroptosis
SCD1 inhibitors: rapamycin (mTOR)	SCD1/SREBP1 axis	Lower MUFA, restores peroxidizable substrate; reverse resistance	Preclinical/clinical (rapamycin approved other indications)
ML385	NRF2	Inhibits oncogenic NRF2; re-sensitizes to ferroptosis	Preclinical
Elesclomol	Copper ionophore (FDX1)	ROS/cuproptosis; crosstalk with ferroptosis	Clinically tested (other endpoints)

## Data Availability

Not applicable.
